# The Effect of Acid Hydrolysis on the Pickering Emulsifying Capacity of Tartary Buckwheat Flour

**DOI:** 10.3390/foods13101543

**Published:** 2024-05-15

**Authors:** Shijie Zhang, Changsheng Guo, Benguo Liu

**Affiliations:** School of Food Science, Henan Institute of Science and Technology, Xinxiang 453003, China; zhangshijie1107@163.com (S.Z.); guo1186958122@163.com (C.G.)

**Keywords:** acid hydrolysis, Tartary buckwheat flour, Pickering emulsion, mechanical properties

## Abstract

The effect of sulfuric acid hydrolysis on the Pickering emulsifying capacity of Tartary buckwheat flour (TBF) rich in starch was evaluated for the first time. The results indicate that the sulfuric acid concentration and hydrolysis time had a significant impact on the Pickering emulsifying capacity of acid-hydrolyzed Tartary buckwheat flour (HTBF). A low sulfuric acid concentration (1–2 mol/L) could reduce the particle size of HTBF, but it also decreased the Pickering emulsifying ability. At a sulfuric acid concentration of 3 mol/L, appropriate treatment time (2 and 3 days) led to particle aggregation but significantly improved wettability, thereby resulting in a rapid enhancement in emulsifying capacity. Under these conditions, the obtained HTBF (HTBF-D2-C3 and HTBF-D3-C3) could stabilize medium-chain triglyceride (MCT)-based Pickering high-internal-phase emulsions (HIPEs) with an oil-phase volume fraction of 80% at the addition amounts (*c*) of ≥1.0% and ≥1.5%, respectively. Its performance was significantly superior to that of TBF (*c* ≥ 2.0%). Furthermore, at the same addition amount, the droplet size of HIPEs constructed by HTBF-D3-C3 was smaller than that of HTBF-D2-C3, and its gel strength and microrheological performance were also superior to those of HTBF-D2-C3, which was attributed to the higher wettability of HTBF-D3-C3. The findings of this study can facilitate the in-depth application of Tartary buckwheat and provide references for the development of novel Pickering emulsifiers.

## 1. Introduction

The modification of starch includes physical modification, chemical modification, enzymatic modification, and compound modification [1]. Acid hydrolysis is a commonly used method in the chemical modification of starch, whereby starch is immersed in a solution of a certain concentration of acid. Under the action of the acid solution, starch granules are gradually broken down, thereby altering the physicochemical properties of starch [1]. Acid hydrolysis can increase the gelatinization temperature of starch, improve starch solubility, and enhance starch emulsifying capacity [2,3,4]. Singh and Ali found that hydrochloric acid and nitric acid had the highest hydrolysis efficiency in preparing acid-hydrolyzed starch, followed by sulfuric acid and phosphoric acid [5]. Królikowska et al., investigated the effect of acid hydrolysis on the structure and functions of starch–oleic acid mixture and found that with the increase in acid treatment time, the final viscosity and thermal paste viscosity of the starch–fatty acid mixture gradually declined [6]. Fouladi and Nafchi found that acid hydrolysis treatment reduced the relative molecular weight of sago starch, decreased its amylopectin content, and facilitated subsequent etherification modification [7].

Pickering high-internal-phase emulsion (HIPE) is an emulsion with a dispersed-phase volume fraction exceeding 74% formed by using solid particles instead of traditional emulsifiers in emulsions. Compared with traditional HIPEs, Pickering HIPEs offer advantages such as high stability, low production costs, high safety, and environmental friendliness. Pickering HIPEs based on food-derived proteins and polysaccharides have attracted widespread attention as the most promising solid fat substitutes and carriers for functional ingredient delivery. However, these food-derived polymers are highly hydrophilic and have poor emulsifying properties, requiring acid hydrolysis, esterification, cross-linking, and other pretreatments to enhance their Pickering emulsifying ability [8]. Recent studies have shown that acid-hydrolyzed starch can be used for the construction of Pickering emulsions [9,10].

Tartary buckwheat is a dicotyledonous crop of Polygonaceae which is rich in starch, protein, dietary fiber, and vitamins [11]. It has high nutritional and medicinal value, with the starch content in its seeds accounting for over 70% of the dry weight [11]. Xiao et al., evaluated the influence of heat–moisture treatment on the structure and functions of buckwheat flour and in vitro simulated digestion [12]. Liu et al., studied the effects of high-hydrostatic-pressure (HHP) treatment on the digestibility, physicochemical properties, and texture characteristics of buckwheat starch and found that it could enhance the resistance of starch [13]. Zhang et al., reported the processing characteristics of buckwheat bran flour/wheat flour blend powder and clarified the impact of adding buckwheat bran flour on the appearance, structure, and digestion of steamed bread [14], and Bist et al., found that buckwheat bran flour had Pickering emulsifying ability [15]. Currently, there is no systematic research on the preparation and Pickering emulsifying ability of acid-hydrolyzed Tartary buckwheat flour (HTBF). In view of this, this study intends to clarify the influence of sulfuric acid concentration and hydrolysis time on the Pickering emulsifying ability of HTBF, in order to promote the in-depth application of Tartary buckwheat and provide reference for the development of new Pickering emulsifiers.

## 2. Materials and Methods

### 2.1. Materials and Chemicals

Tartary buckwheat was obtained from the Crop Science Research Institute of Shanxi Agricultural University (Taiyuan, China) and was harvested in 2021. Medium-chain triglyceride (MCT) was obtained from Shanghai Yuanye Technology Co., Ltd. (Shanghai, China). Other reagents were of analytical grade.

### 2.2. Preparation of Hydrolyzed Tartary Buckwheat Flour (HTBF)

Tartary buckwheat flour (TBF) came from our previous research [14]. The moisture, starch, and protein contents of TBF were 10.78 ± 0.03%, 81.94 ± 0.15%, and 8.07 ± 0.06%, respectively. HTBF was prepared according to the method by Gonzalez et al. [16]. A total of 15 g of TBF and 100 mL of sulfuric acid solution at concentrations of 1 mol/L, 2 mol/L, and 3 mol/L were mixed and oscillated at 40 °C at 150 r/min for 1, 2, and 3 days. After centrifugation at the end of the reaction, the precipitate was washed repeatedly with distilled water to neutrality and subsequently lyophilized in an Alpha 1-2 freezing drier (Christ, Hagen, Germany) to obtain HTBF. The obtained samples were named HTBF-D1-C1, HTBF-D1-C2, HTBF-D1-C3, HTBF-D2-C1, HTBF-D2-C2, HTBF-D2-C3, HTBF-D3-C1, HTBF-D3-C2, and HTBF-D3-C3 according to hydrolysis time (D) and sulfuric acid concentration (C). Their color parameters (L*, a*, and b*) were measured by using a CR-400 colorimeter (KonicaMinolta, Osaka, Japan).

### 2.3. Determination of Particle Size

Following the method proposed by Xia et al. [17], the sample (2.0 g) was dispersed in the dispersion tank of a BT-9300H laser particle size analyzer (BETTER, Dandong, China), and the particle size distribution was recorded when the shading rate was stable at 15%-16%. The refractive index of the dispersant was 1.33, and the refractive index of the sample was 1.53.

### 2.4. Determination of Interfacial Tension

Based on the report by Alteraifi et al. [18], the tip containing the 10 mg/mL sample solution was inserted into a quartz vessel containing MCT, and 12 μL of the sample solution was dispensed. The changes in the droplet over 20 s were recorded at a rate of 1 frame/s by using a Theta Lite optical contact angle measurement instrument (Biolin Scientific, Stockholm, Sweden), and the interfacial tension was calculated by using OneAttension 4.0.4 software (Biolin Scientific, Stockholm, Sweden).

### 2.5. Determination of Contact Angle

According to the report by Kirk et al. [19], the suppressed sample disc (diameter of 2 cm and thickness of 2 mm) was placed at the bottom of a quartz vessel containing MCT. By using a syringe, 12 μL of water was dropped onto the surface of the sample. The changes in the droplet were recorded at 1 frame/s for 20 s by using a Theta Lite optical contact angle measurement instrument (Biolin Scientific, Stockholm, Sweden). The contact angle was calculated by using OneAttension software (Biolin Scientific, Stockholm, Sweden).

### 2.6. Preparation of Pickering HIPEs

Referring to the method by Wang et al. [20], MCT was used as the oil phase, while a certain amount of sample was dispersed in water as the aqueous phase. They were mixed at an oil-phase volume fraction (*φ*) of 80% and homogenized at 12,000 rpm for 3 min to prepare Pickering HIPEs with a sample concentration (*c*) ranging from 1.0% to 2.5% (*w*/*v*). The stability of the HIPEs was determined based on the inverted bottle method.

### 2.7. Microscopic Observation of Pickering HIPEs

Pickering HIPEs were obtained by using HTBF-D2-C3 and HTBF-D3-C3 as emulsifiers under the conditions of *c* = 1.5, 2.0, and 2.5% and *φ* = 80%. The appearance was observed by using a BH200P microscope (Hengping, Shanghai, China), and the size distribution of the oil droplets was measured by using a laser particle size analyzer [21].

### 2.8. Gel Strength Measurement of Pickering HIPEs

Pickering HIPEs were obtained with HTBF-D2-C3 and HTBF-D3-C3 as emulsifiers at *c* = 1.5, 2.0, and 2.5% and *φ* = 80%. Their gel strength values were analyzed with a TA-XT plus texture analyzer equipped with a P0.5 probe (Stable Micro Systems, Surrey, UK) [22]. The measurement parameters were as follows: trigger force, 2 g; compression distance, 25 mm; probe speed, 1.0 mm/s.

### 2.9. Microrheological Determination of Pickering HIPEs

Pickering HIPEs were obtained with HTBF-D2-C3 and HTBF-D3-C3 as emulsifiers at *c* = 1.5, 2.0, and 2.5% and *φ* = 80%. The 20 mL newly prepared Pickering HIPEs were placed into the Rheolaser LAB6 microrheological analyzer (Formulation, France), and the evolution of the macroscopic viscosity index (MVI) and the elastic index (EI) over time were recorded [23].

### 2.10. Statistical Analysis

The experimental results were presented as means ± standard deviations (*n* = 3). Data processing was conducted by using SPSS 18.0 software, and differences were considered statistically significant when *p* < 0.05.

## 3. Results and Discussion

### 3.1. Appearance and Yield of HTBF

**Figure 1:**
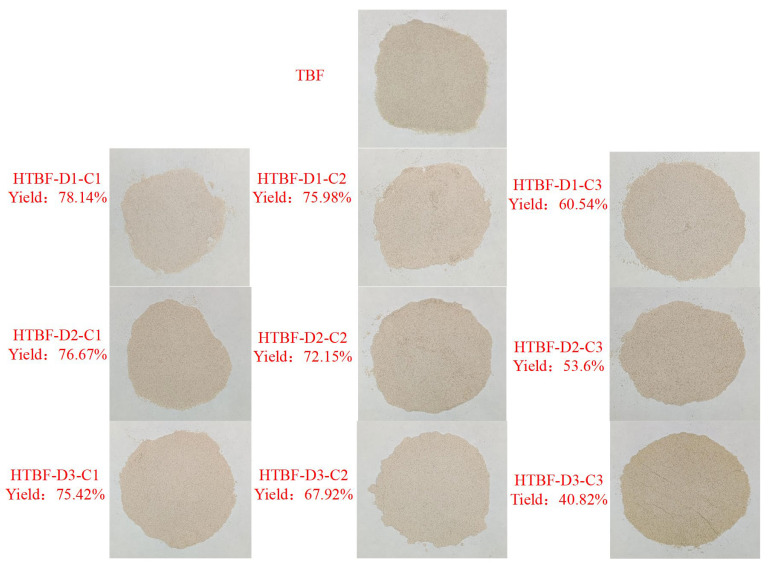
Appearance and yield of HTBF.

This study utilized a method of crushing combined with screening to remove the husk and bran of Tartary buckwheat, obtaining its endosperm. Therefore, the TBF obtained was rich in starch, with dry basis starch content as high as 91.84%, approaching the starch content of corn and potato starch [24]. As shown in Figure 1 and Table 1, compared with TBF, the brightness of samples treated with 3 mol/L sulfuric acid for 2 and 3 days (HTBF-D2-C3 and HTBF-D3-C3) decreased distinctly, accompanied by a shift towards red and yellow hues. HTBF-D3-C3 exhibited the lowest yield (40.82%). This may have been due to excessive acid hydrolysis at this time, leading to the significant destruction of starch, protein, and other components, resulting in a darker appearance of the particles. Tang et al., also found that acid hydrolysis could significantly alter the morphology and particle size distribution of KGM particles [25].

### 3.2. Particle Size Analysis

**Figure 2:**
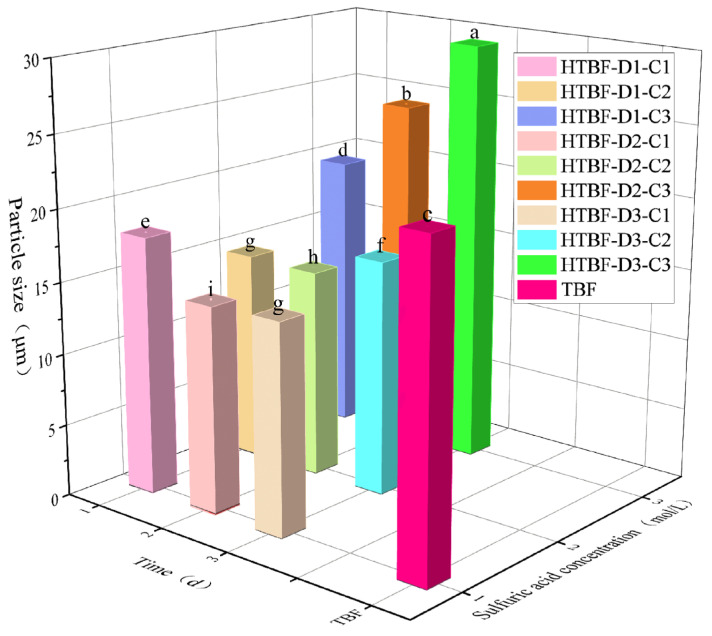
Particle size of TBF and HTBF (different letters in the same figure indicate significant differences (*p* < 0.05)).

When the sulfuric acid concentration was 1 or 2 mol/L, the size of HTBF gradually decreased with the extension of hydrolysis time (Figure 2). This was because sulfuric acid first acted on the amorphous region of starch granules, causing the starch granules to shrink. Zuo et al., also found that the particle size of corn starch after acid hydrolysis treatment was smaller than that of natural corn starch [26]. However, when the sulfuric acid concentration was 3 mol/L, with the increase in hydrolysis time, the size of HTBF gradually increased. The particle size of HTBF-D2-C3 and HTBF-D3-C3 was significantly larger than other HTBF samples, with the particle size of HTBF-D3-C3 reaching its maximum value (31.05 μm). This was due to the high damage degree of starch particles under the action of acid, leading to mutual adhesion during the drying process and resulting in aggregation.

### 3.3. Interface Tension Analysis

**Figure 3:**
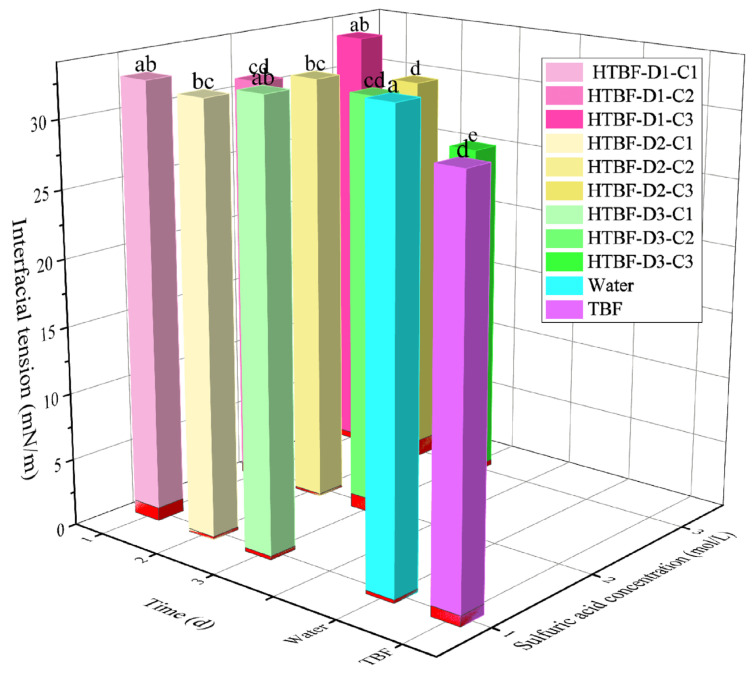
Effects of TBF and HTBF on the oil/water interfacial tension (different letters in the same figure indicate significant differences (*p* < 0.05)).

The interfacial properties of emulsifiers are related to their emulsifying ability and stability [27]. The influence of acid hydrolysis on the interfacial tension of buckwheat flour was analyzed in this study (Figure 3). TBF could significantly reduce the oil/water interfacial tension. Zhang et al., also reported that buckwheat flour had Pickering emulsifying ability, which could stabilize HIPEs with *φ* = 80%. This was mainly attributed to its high protein content [15]. After treating the TBF samples with 1 mol/L and 2 mol/L sulfuric acid for 1–3 days, the corresponding interfacial tension showed an increasing trend, which could be due to the hydrolysis of proteins and a decrease in particle emulsifying ability under these conditions. However, when TBF was treated with 3 mol/L sulfuric acid, as the treatment time increased, the emulsifying effect of the particles rapidly improved. Both HTBF-D2-C3 (30.04 mN/m) and HTBF-D3-C3 (25.63 mN/m) could reduce the interfacial tension, which was attributed to the acid treatment enhancing the emulsifying ability of starch particles. Guo et al., also found that the ability of acorn starch to reduce the oil/water interfacial tension rapidly declined after being treated with 3.0 and 4.0 mol/L sulfuric acid for 2 days [28].

### 3.4. Contact Angle Analysis

**Figure 4:**
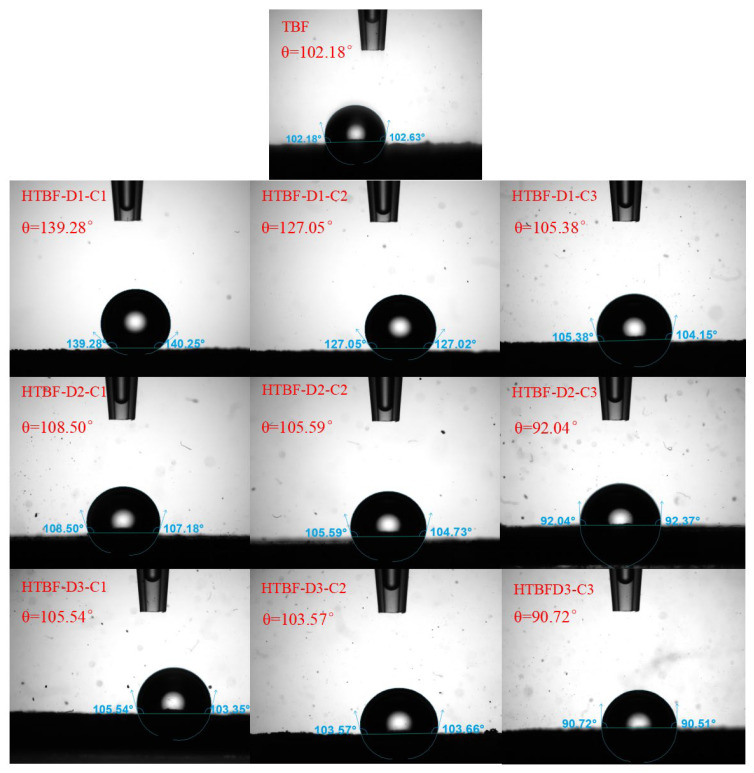
Contact angles of TBF and HTBF.

Starch has a certain degree of wettability, which can stabilize Pickering emulsions [29], and the contact angle test can reflect the wettability of starch. In this study, the influence of acid hydrolysis on the contact angle of HTBF was also evaluated. The contact angle of TBF was 102.18° (Figure 4), exhibiting hydrophobicity. However, after treating TBF samples with 1 mol/L and 2 mol/L sulfuric acid for 1–3 days, the contact angle of HTBF was higher than that of TBF, indicating an increase in hydrophobicity, possibly due to the loss of hydrophilic proteins. However, when the sulfuric acid concentration increased to 3 mol/L, with the extension of processing time, the hydrophilicity of THBF rapidly increased. The contact angles of HTBF-D2-C3 and HTBF-D3-C3 were both close to the optimal theoretical contact angle value (90°) of Pickering emulsifier particles [30], consistent with the results of interfacial tension testing. The contact angle and interfacial tension results indicated that the Pickering emulsifying ability of HTBF-D2-C3 and HTBF-D3-C3 was expected to be higher than that of TBF.

### 3.5. Formation of Pickering HIPEs

**Figure 5:**
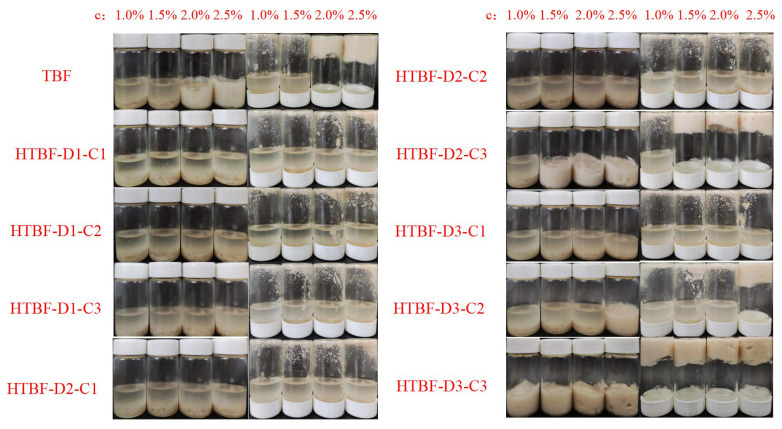
The appearance of the Pickering emulsions developed by TBF and HTBF at φ = 80%.

In this study, MCT-based Pickering HIPEs based on TBF and HTBF were constructed under the conditions of *φ* = 80% and *c* = 1.0–2.5% (Figure 5). TBF could only fabricate stable Pickering HIPEs at *c* = 2.0% and 2.5%; HTBF-D2-C3 could stabilize Pickering HIPEs at *c* = 1.5%, 2.0%, and 2.5%; and HTBF-D3-C3 could construct Pickering HIPEs at *c* = 1.0–2.5%. This indicated the ranking of emulsifying ability as HTBF-D3-C3 > HTBF-D2-C3 > TBF, which was consistent with the results of the contact angle and interfacial tension assays. Fonseca-Florido et al., also observed that hydrochloric acid could enhance the emulsifying capacity of wax cassava starch [31].

### 3.6. Microscopic Analysis of Pickering HIPEs

**Figure 6:**
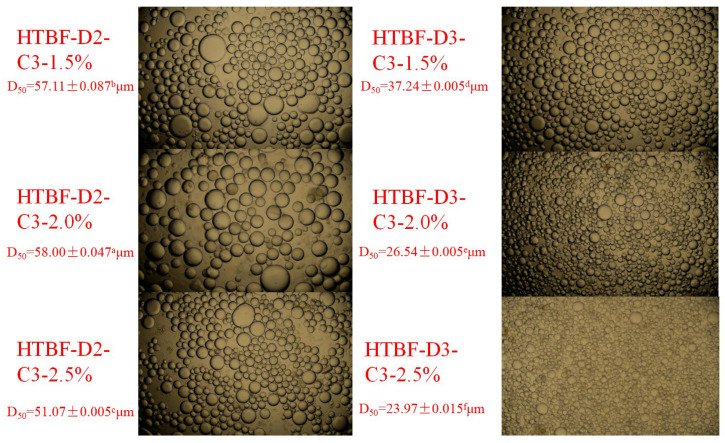
The microscopic analysis of the Pickering emulsions developed by HTBF-D2-C3 and HTBF-D3-C3 at *φ* = 80% and *c* = 1.5, 2.0, and 2.5%.

With HTBF-D2-C3 and HTBF-D3-C3 as emulsifiers, MCT-based Pickering HIPEs were constructed at *c* = 1.5%, 2.0%, and 2.5%. Figure 6 exhibits the microscopic analysis of these HIPEs. Under the same conditions, the droplet size of Pickering HIPEs developed by HTBF-D3-C3 was smaller than that of HTBF-D2-C3, which confirmed that the emulsifying ability of HTBF-D3-C3 was superior to that of HTBF-D2-C3. In addition, for the same type of HTBF, with the increase in *c*, its emulsion droplet size gradually decreased. This was because increasing the emulsifier dosage could meet higher oil–water interface requirements, resulting in smaller droplet size. Saari et al., also observed similar phenomena in their investigation on Pickering HIPEs stabilized by quinoa/waxy maize starch [32].

### 3.7. Gel Strength Analysis of Pickering HIPEs

**Figure 7:**
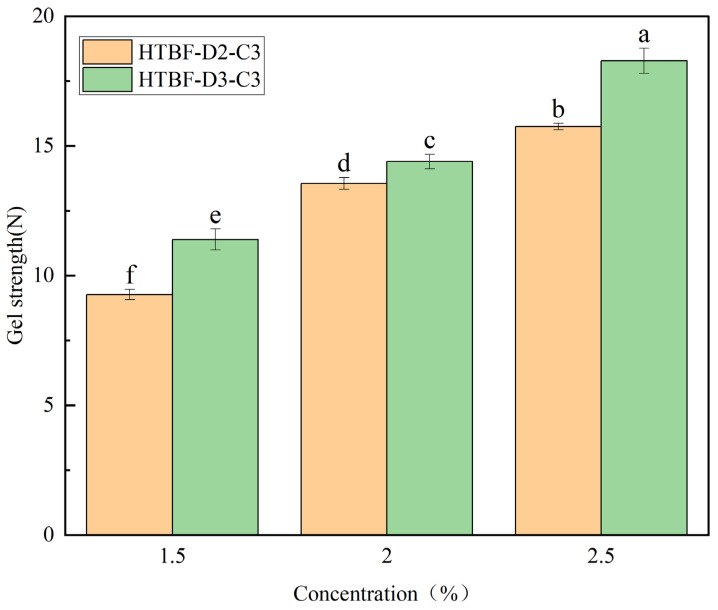
The gel strength of the Pickering emulsions developed by HTBF-D2-C3 and HTBF-D3-C3 at *φ* = 80% and *c* = 1.5, 2.0, and 2.5% (different letters in the same figure indicate significant differences (*p* < 0.05)).

As shown in Figure 7, with the increase in HTBF addition, the gel strength of the resulting Pickering HIPEs gradually ascended. At the same addition amount, the gel strength of the gel formed by HTBF-D3-C3 was significantly higher than that of HTBF-D2-C3. Jia et al., found that the addition of emulsifiers had a positive effect on gel strength [33], while Geng et al., also observed a positive correlation between the mechanical parameters of emulsions and the addition amount of proanthocyanidin particles [34]. This is because starch has a viscosity effect: with the increase in addition amount, the viscosity of the system is higher, and the oil droplets are more difficult to merge. Furthermore, at the same addition concentration, the gel strength of the sample developed by HTBF-D3-C3 was higher than that of HTBF-D2-C3. The strong emulsifying ability of TBF-D3-C3 resulted in smaller droplet size and a more compact system, which coincided with the microscopic analysis results.

### 3.8. Microrheological Analysis of Pickering HIPEs

**Figure 8:**
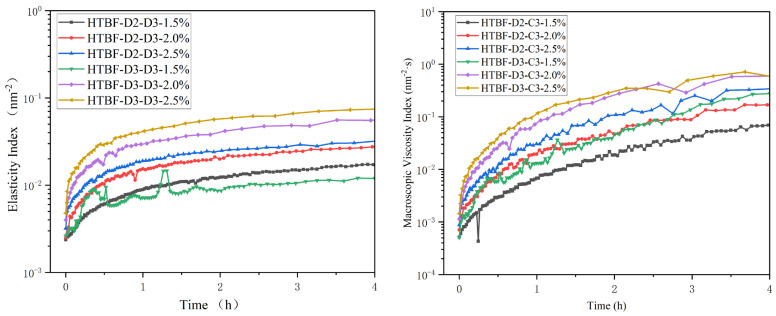
The elasticity index (EI) and macroscopic viscosity index (MVI) of the Pickering emulsions developed by HTBF-D2-C3 and HTBF-D3-C3 at *φ* = 80% and *c* = 1.5, 2.0, and 2.5%.

Microrheology is a method of studying rheology by using diffusing-wave spectroscopy (DWS), where the measured values of EI and MVI can reflect the strength and viscosity of emulsion systems, respectively, in the zero-shear state [35]. As shown in Figure 8, the EI value of the system was positively correlated with the MVI value and *c* value. The greater the amount of emulsifier added, the greater the viscosity and elasticity of the gel. Lv et al., also reported that the MVI and EI values of the system increased with the addition of esterified tigernut starch [36]. When the addition amount was the same, the MVI value of the gel with HTBF-D3-C3 was higher than that of HTBF-D2-C3 at the same time. This was attributed to the smaller droplet size of the emulsion stabilized by HTBF-D3-C3, resulting in a more compact system with higher viscosity, which coincided with the gel strength result. This further confirmed that the emulsifying ability of HTBF-D3-C3 was higher than that of HTBF-D2-C3. The structure and physicochemical properties of the emulsions were closely related to the emulsifier. Wang et al., found significant differences in the droplet size of the Pickering emulsion constructed by using ammonia-treated waxy maize starch (NWS) and native corn starch (NCS) [20].

## 4. Conclusions

Acid hydrolysis with different concentrations of sulfuric acid and treatment times had a significant impact on the Pickering emulsifying capacity of Tartary buckwheat flour (TBF), resulting in the formation of HTBF (acid-hydrolyzed Tartary buckwheat flour). It was demonstrated that the sulfuric acid concentration and hydrolysis time directly affected the particle size of HTBF and its emulsifying capacity. Lower sulfuric acid concentrations resulted in smaller particles, but with lower emulsifying capacity, while higher concentrations, combined with appropriate treatment times, led to particle aggregation and a significant improvement in emulsifying capacity. HTBF produced under specific acid hydrolysis conditions was able to stabilize high-internal-phase emulsions (HIPEs) based on medium-chain triglyceride (MCT) with an oil-phase volume fraction of 80%, significantly outperforming TBF. Furthermore, it was observed that the emulsion constructed with HTBF under specific conditions exhibited superior properties regarding droplet size, gel strength, and microrheological performance, attributed to the higher wettability of HTBF under these conditions. These findings represent a significant contribution to the understanding of the emulsifying properties of Tartary buckwheat flour and provide important guidelines for the development of novel Pickering emulsifiers with potential applications in various industries.

## Figures and Tables

**Table 1:** The color parameters of HTBF.

Sample	L*	a*	b*
TBF	79.31 ± 0.81 ^cd^	1.08 ± 0.08 ^h^	14.55 ± 0.06 ^e^
HTBF-D1-C1	82.69 ± 0.22 ^a^	3.50 ± 0.10 ^f^	14.52 ± 0.07 ^e^
HTBF-D1-C2	81.22 ± 0.16 ^b^	3.60 ± 0.26 ^e^	13.91 ± 0.11 ^f^
HTBF-D1-C3	78.19 ± 0.54 ^d^	3.79 ± 0.15 ^c^	14.07 ± 0.07 ^f^
HTBF-D2-C1	79.63 ± 0.37 ^c^	4.06 ± 0.06 ^b^	16.76 ± 0.07 ^b^
HTBF-D2-C2	78.59 ± 0.64 ^cd^	3.68 ± 0.26 ^de^	15.49 ± 0.53 ^d^
HTBF-D2-C3	76.66 ± 0.21 ^e^	3.64 ± 0.03 ^de^	16.34 ± 0.03 ^c^
HTBF-D3-C1	78.79 ± 0.43 ^cd^	4.57 ± 0.02 ^a^	16.20 ± 0.08 ^c^
HTBF-D3-C2	78.41 ± 0.19 ^d^	3.66 ± 0.05 ^de^	14.64 ± 0.14 ^e^
HTBF-D3-C3	75.28 ± 0.39 ^f^	3.06 ± 0.04 ^g^	18.21 ± 0.08 ^a^

Different letters in the same column indicate significant differences (*p* < 0.05).

## Data Availability

The original contributions presented in the study are included in the article, further inquiries can be directed to the corresponding author.
